# Ovarian Sertoli–Leydig cell tumors: MRI findings and pathological correlation

**DOI:** 10.1186/1757-2215-6-73

**Published:** 2013-10-26

**Authors:** Song-Qi Cai, Shu-Hui Zhao, Jin-Wei Qiang, Guo-Fu Zhang, Xue-Zhen Wang, Li Wang

**Affiliations:** 1 Department of Radiology, Jinshan Hospital, Shanghai Medical College, Fudan University, Shanghai 201508, China; 2 Department of Radiology, Obsterics & Gynecology Hospital, Shanghai Medical College, Fudan University, Shanghai 200011, China; 3 Department of Pathology, Jinshan Hospital, Shanghai Medical College, Fudan University, Shanghai 201508, China

**Keywords:** Ovary, Sertoli-Leydig cell tumors, Magnetic resonance imaging, Pathology

## Abstract

**Background:**

To investigate the magnetic resonance imaging (MRI) characteristics of ovarian Sertoli-Leydig cell tumors (SLCT).

**Methods:**

The clinical, MRI and pathological findings of five cases of SLCT were reviewed retrospectively. MRI appearances of tumors including laterality, shape and size, architecture, wall, septa and vegetation, signal intensity and contrast-enhancement pattern were evaluated and correlated with pathological findings.

**Results:**

Two tumors were solid which appeared as low signal intensity on T1-weighted imaging (T1WI) and moderate on T2-weighted imaging (T2WI) with multiple small cysts in one of them. The remaining three SLCT were multilocular cystic with the irregularly thickened wall and septa, and with solid area and mural nodules in one of them. The cystic components had the same signal intensity as urine. All the solid components were intensely enhanced after administration of contrast medium. All five tumors were pathologically intermediate differentiation and at FIGO stage I.

**Conclusions:**

SLCT demonstrate variable MRI morphological appearances. However, the irregularly thickened wall and septa, the moderate T2WI signal intensity and obvious enhancement in the solid components are three MRI features.

## Background

Sertoli-Leydig cell tumors (SLCT) of ovary are a rare type but well defined clinicopathologic entity of sex cord-stromal tumors, accounting for less than 0.5% of ovarian neoplasms [1]. These tumors are histopathologically characterized by the presence of variable proportions of Sertoli and Leydig cells. Most SLCT occur in young women and are at stage I (International Federation of Gynecology and Obstetrics, FIGO) [2]. Conservative surgery is acceptable for young patients who wish to preserve fertility without compromising 5-year disease-specific survival [3]. Some clinicopathological studies of SLCT have been reported. To our knowledge, however, only three imaging case reports have been published [4-6]. The present study described the magnetic resonance imaging (MRI) appearances of five patients with SLCT by correlated with pathological findings, with the aim to be familiar with the imaging appearances of this entity and improve the accuracy of preoperative diagnosis.

## Methods

### Patients

This retrospective study was approved by the institutional review boards of our hospitals and informed consent was waived. Patients with suspected ovarian tumor were enrolled in an ovarian tumor MRI study project from February 2008 to November 2012 at Jinshan Hospital and Obstetrics & Gynecology Hospital of Fudan University. Among 530 cases proven by surgery and histopathology, a total of five patients with SLCT were found.

### MRI scan and analysis

All patients were performed with 1.5-T MRI scanners (Avanto or Symphony, Siemens, Erlangen, Germany) using a torso phased-array coil with patient supine and free breathing. The following sequences were obtained: spin echo (SE) axial T1-weighted imaging (T1WI ) [time of repetition (TR)/time of echo (TE), 340 ms/10 ms]; fast low-angle shot (FLASH) 2D T1WI with fat saturation (TR/TE, 196 ms/2.9 ms); turbo spin echo (TSE) T2-weighted imaging (T2WI) with and without fat saturation (TR/TE, 8000 ms/83 ms and 4000 ms/98 ms) respectively. Sagittal and coronal TSE T2WI (TR/TE, 8000 ms/98 ms) were also obtained. The contrast-enhanced FLASH 2D T1WI with fat saturation (TR/TE, 196 ms/2.9 ms) was performed in the axial, sagittal and coronal planes right after intravenous administration of Gadopentetate dimeglumine (Gd-DTPA, Magnevist; Bayer Schering, Guangzhou, China) at a dose of 0.1 mmol/kg of body weight and a rate of 2 ml/s. The scanning parameters were as follows: 5-mm slice thickness, 1.5-mm gap, 256 × 256 matrix, 20–25 cm × 34 cm field of view and four excitations. The scanning range was from the inferior pubic symphysis to the renal hilum and was extended beyond the dome of tumor in the cases with huge masses.

The MR images were reviewed independently by two radiologists who specialized in gynecological imaging. Their interpretations were arrived at by consensus. The following features were evaluated: (a) laterality, shape and size; (b) cystic or solid components; (c) thickness of wall and septa (considered thin when ≤3 mm and thick when > 3mm); (d) vegetations; (e) signal intensity (the signal similar to inner, outer myometrium and fat was considered low, moderate and high, respectively); (f) contrast-enhancement (the enhancement of solid component less than or equal to that of innermyometrium, between inner and outer myometrium, and equal to or greater than outer myometrium was considered mild, moderate or obvious, respectively). The predominant signal was taken to represent the tumor’s signal, where the tumor was heterogeneous.

### Pathological analysis

Histopathological examination was performed by a radiologist and a pathologist, both were familiar with or specialized in gynecological pathology. After determining the shape and size of the tumors, the gross specimens were dissected into 1 to 5 cm sections. The tumors were analyzed including: shape, size, architecture, the presence of loculus and the nature of the intracystic content; thickness of wall and septa. The specimens were fixed in 10% formalin solution, embedded in paraffin, sectioned, stained with hematoxylin and eosin (H&E), immunohistochemical inhinbin-α, EMA and cytokeratin 7. A correlation between imaging and histopathological findings was performed together by a pathologist and a radiologist.

## Results and discussion

Only one of five patients presented with deepening of the voice and increasing of facial hair. Three cases presented with abdominal swelling. The remaining case was asymptomatic and found during the routine physical examination. Serum testosterone level (normal, 0.15-0.5 ng/ml) elevated in two cases, and CA125 level (normal, < 35 U/ml) elevated in one. Radical surgery was performed in two cases, unilateral salpingo-oophorectomy in one case, and cystectomy in the other two. The clinical manifestations of five cases of SLCT are listed in Table 1.

**Table 1:** The clinical manifestations of five cases of Sertoli-Leydig cell tumors

**Case no.**	**Age**	**Symptoms**	**CA125**	**Testo**	**Surgery**	**Pathological differentiation**	**FIGO stage**
1	30	Virilization	11.8	5.9	LAUC	Intermediately	Ia
2	46	None	\	\	TRS^#^	Intermediately	Ia
3	22	Abdominal swelling	92.9	\	LAUC	Intermediately	Ia
4	56	Abdominal swelling	24	1.51	TRS	Intermediately *	Ia
5	25	Abdominal swelling	\	\	LAUS	Intermediately	Ia

CA125 (normal, <35 U/ml); Testo = Testosterone (normal, 0.15-0.5 ng/ml); LAUC = Laproscopically assisted unilateral cystectomy; LAUS = Laproscopically assisted unilateral salpingo-oophorectomy. TRS = Transabdominal radical surgery (total hysterectomy and bilateral salpingo-oophorectomy); # TRS followed by chemotherapy. * Intermediately differentated with mucinous heterologous.

**Figure 1:**
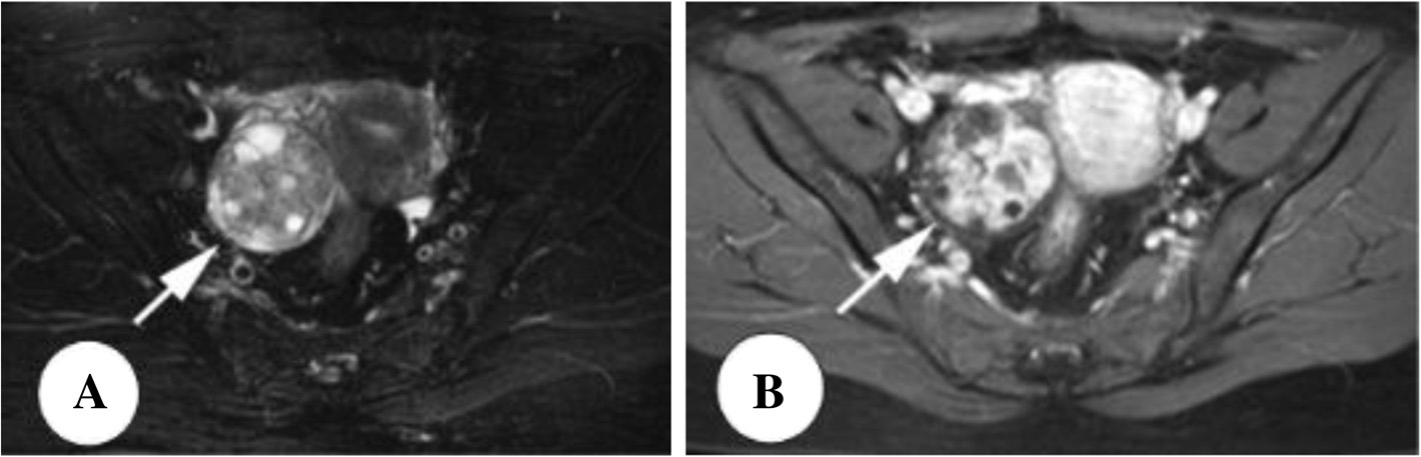
**Sertoli-Leydig cell tumor in a 46-year-old woman without symptoms. (A)** Axial T2-weighted image with fat saturation shows a solid mass with multiple small cysts (arrow). **(B)** The solid components are the moderate signal intensity and obviously enhanced after the administration of contrast medium.

**Figure 2:**
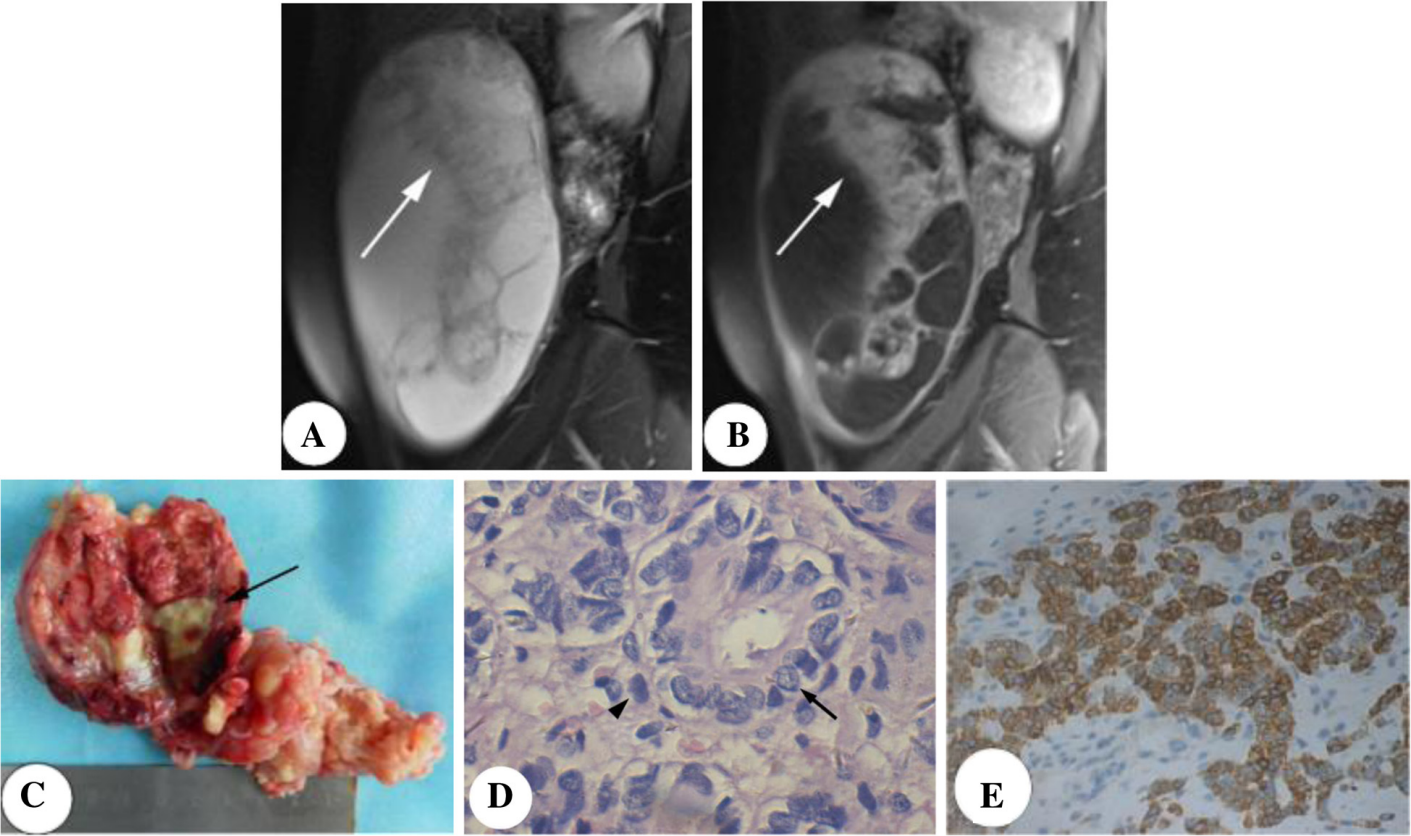
**Sertoli-Leydig cell tumor in a 22-year-old woman with irregular menses and abdominal swelling.** Sagittal T2-weighted image demonstrates a huge multilocular cystic mass with solid area and mural nodules (white arrow), which are the moderate signal intensity **(A)** and enhanced intensely after the administration of contrast medium **(B)**. **(C)** Grossly, the tumor shows an encapsulated cystic mass (fluid has been removed) with reddish solid area and yellowish nodules (black arrow). **(D)** Microscopically, Leydig cells (arrowhead) are sparse in the background of Sertoli cells, which arrange in closed tubules (black arrow). The sertoli cells have abundant vacuolated cytoplasm filled with lipid (H&E×400). **(E)** The sex cord areas stain strongly for α-inhibin (α-inhibin stain ×200).

**Figure 3:**
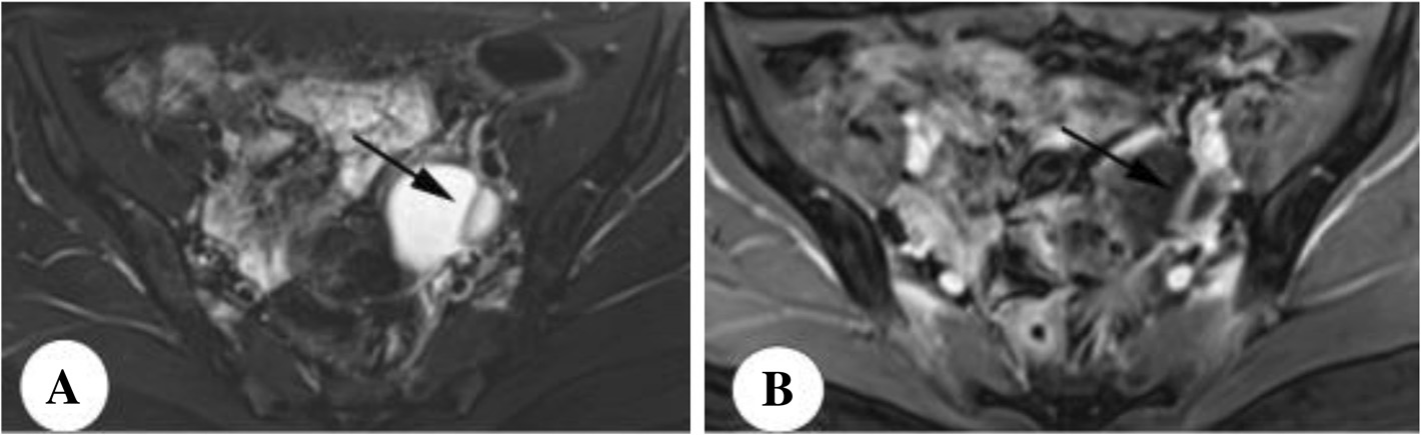
**Sertoli-Leydig cell tumor in a 25-year-old woman with irregular menses.** Axial T2-weighted image with fat saturation **(A)** shows a bilocular cystic mass with thickened septa and wall (arrow), which are intensely enhanced **(B)**.

MRI findings of five cases of SLCT are summarized in Table 2. Four tumors occurred in left ovary and the remaining one in right ovary; two tumors were solid with the round shape and demarcatedmargin (Figure 1). Multiple small cysts were found in one of the solid tumors. The signal intensities of solid masses were low on T1WI and moderate on T2WI. Three tumors were multilocular cystic (2, 10 and 12 loculi respectively) with irregularly thickened wall and septa, and one of them had solid area and mural nodules with a broad base. The signal intensities of the solid area (Figure 2A) and nodules were the same as those of the solid tumors. The signal intensities of the cystic components were the same as those of the urine. After administration of contrast medium, the cystic wall and septa (Figure 3B), solid area and nodules, and solid masses were enhanced intensely which was iso- to hyperintense to that of outer myometrium. Small amount of ascites were found in three cases. However, no extension to the pelvic organs, peritoneal inplants and enlarged lymph nodes was observed.

**Table 2:** The MRI findings of five cases of Sertoli-Leydig cell tumors

**Case no**	**Site**	**Size (cm**^**3**^**)**	**Components**	**SI (solid)**		**SI (cystic)**		**Enhancement (solid)**
				**T1WI**	**T2WI**	**T1WI**	**T2WI**	
1	Left	6.6×6.1×6.2	Purely solid	Low	Moderate	\	\	Intense
2	Right	4.7×5.0×4.2	Mainly solid^#^	Low	Moderate	Low	High	Intense
3	Left	25×20×38	Mainly cystic*	Low	Moderate	Low	High	Intense
4	Left	20×15×15	Purely cystic	Low	Moderate	Low	High	Intense
5	Left	3.7×4.3×4.3	Purely cystic	Low	Moderate	Moderate/low	High	Intense

SI = Signal Intensity; ^#^ Mainly Solid with multiple small cysts; * Mainly Cystic with intracystic solid area and mural nodules.

The macroscopic appearances confirmed the MRI morphological findings (Figure 2C). Histopathologically, Leydig cells had the eosinophilic cytoplasm, scattering in the background of numerous Sertoli cells, some of which have abundant vacuolated cytoplasm filled with lipid (Figure 2D). A few cords and poorly developed tubules of Sertoli cells were found, with some atypia. The immunohistochemical analysis showed that sex cord-stromal cells stained intensely positive for inhinbin-α and negative for the cytokeratin 7 receptors. The mucinous components expressed strong cytoplasmic positivity for cytokeratin 7.

SLCT are the most common virilizing ovarian tumors and originally named as arrhenoblastomas or androblastomas. However, some tumors are nonfunctioning, and even estrogenic. Therefore, World Health Organization renamed them as “Sertoli-Leydig cell tumors” according to their histopathological features [7]. Young et al. [2] reviewed the clinicopathological data of 207 patients with SLCT. Their ages ranged from 2 to 75 years (average 25 years), with about 75% of the patients being 30 years or younger and less than 10% over 50 years; about one-third of the patients presented an elevation of androgen or related symptoms and signs and most of the patients without hormonal effects complained abdominal swelling or pain. Similar findings had also been reported [8-11]. The largest tumor in Young et al.’ series was 51 cm in diameter (average 13.5 cm) and 97.5% of patients were at FIGO stageI, 1.5% at stage II, and 1% at stage III. Tumors usually occured unilaterally, only 1.5% involved both ovaries. Thirty-eight percent of the tumors were solid, 58% solid and cystic, and 4% cystic. Most of SLCT behave in a benign fashion with biologic behavior correlating with degree of differentiation and stage [2,12]. In our series, mean age was 38.5 years. The tumors were intermediately-differentiated and at stage I in all five cases.

Pathologically, SLCT are divided into four subtypes: well- (11%), intermediately- (54%), and poorly-differentiated (13%) and contained heterologous elements (22%). Heterologous elements are various, such as carcinoid, mesenchymal, and mucinous epithelial tissues with the commonest being gastrointestinal-types [13,14]. Retiform pattern presents in 15% of SLCT, and is found only in intermediately and poorly differentiated SLCT, and often occurs in younger women [2,15,16]. SLCT contain Sertoli cells, Leydig cells, gonadal stromal cells and heterologous cells in tumors with heterologous elements. Leydig cells scatter among numerous Sertoli cells, which may arrange into open or closed tubules. The Sertoli cells may have intracytoplasmic lipids, which may explain the yellowish nodules inside encapsulated mass [6,8,17]. And the intracelluar lipid can not be detected by fat saturation. Immunohistochemical staining is positive for alpha-inhibin (sex cord-stromal marker) and positive for cytokeratin 7 for the mucinous elements [1].

In our study, there were two solid and three cystic masses. SLCT showed some MRI features: the irregularly thickened wall and septa, the moderate signal intensity in the solid components on T2WI, which is accordance with previous literatures [6,17], and the obvious enhancement of the solid components after administration of Gd-DTPA. Azuma et al. [5] reported one case of multilocular cystic SLCT, which demonstrated hyperintensity on T1WI and hypointensity on T2WI considering the hemorrhagic nature of the fluid. In our series, however, the signal intensities of the cystic components were the same as those of the urine.

All five SLCT were misdiagnosed in preoperative MRI in our series. Two solid SLCT were misdiagnosed as fibrothecoma. The mild to moderate enhancement in the latter would be helpful in differentiating from SLCT. In addition, solid SLCT should be distinguished from granulosa cell tumors and ovarian carcinomas which often have appearances of estrogen-producing or irregular necrosis. Three cystic tumors were misdiagnosed as cystadenoma. The cystic SLCT shares the similar gross appearances with ovarian cystadenoma due in part to the mucinous heterologous elements [18]. However, the signal intensity in different loculi of cystadenoma is frequently heterogeneous [19]. The presence of intracystic nodules calls for the need of the differentiation of cystic SLCT from borderline/malignant serous or mucinous tumors. In our case, the nodules are of broad base which is different from the narrow base of papillary projections in epithelial tumors [17].

## Conclusions

SLCT are rare and demonstrate variable MRI morphological appearances, they should be considered in young female with virilization, especially in solid or multilocular cystic masses, with an irregularly thickened wall and septa, a moderate signal intensity on T2WI and an obvious enhancement in solid components.

## Competing interests

We declare that we have no conflict of interest.

## Authors’ contributions

Guarantor of integrity of entire study, JWQ; study concepts/study design or definition of intellectual content, JWQ and GFZ; data analysis/data acquisition, SQC and SHZ; literature research, SQC and JWQ; clinical studies, all authors; and manuscript editing SQC and JWQ. All authors read and approved the final manuscript.
